# Integrating cytogenetics and genomics in comparative evolutionary studies of cichlid fish

**DOI:** 10.1186/1471-2164-13-463

**Published:** 2012-09-09

**Authors:** Juliana Mazzuchelli, Thomas David Kocher, Fengtang Yang, Cesar Martins

**Affiliations:** 1 Department of Morphology, Bioscience Institute, UNESP - São Paulo State University, 18618-970, Botucatu, SP, Brazil; 2 Department of Biology, University of Maryland, College Park, MD, 20742, USA; 3 Wellcome Trust Sanger Institute, Wellcome Trust Genome Campus, Hinxton, Cambridge, CB10 1SA, UK

**Keywords:** Cichlidae, Genome evolution, Molecular cytogenetics, Chromosome, Linkage groups, BACs

## Abstract

**Background:**

The availability of a large number of recently sequenced vertebrate genomes opens new avenues to integrate cytogenetics and genomics in comparative and evolutionary studies. Cytogenetic mapping can offer alternative means to identify conserved synteny shared by distinct genomes and also to define genome regions that are still not fine characterized even after wide-ranging nucleotide sequence efforts. An efficient way to perform comparative cytogenetic mapping is based on BAC clones mapping by fluorescence *in situ* hybridization. In this report, to address the knowledge gap on the genome evolution in cichlid fishes, BAC clones of an *Oreochromis niloticus* library covering the linkage groups (LG) 1, 3, 5, and 7 were mapped onto the chromosomes of 9 African cichlid species. The cytogenetic mapping data were also integrated with BAC-end sequences information of *O. niloticus* and comparatively analyzed against the genome of other fish species and vertebrates.

**Results:**

The location of BACs from LG1, 3, 5, and 7 revealed a strong chromosomal conservation among the analyzed cichlid species genomes, which evidenced a synteny of the markers of each LG. Comparative *in silico* analysis also identified large genomic blocks that were conserved in distantly related fish groups and also in other vertebrates.

**Conclusions:**

Although it has been suggested that fishes contain plastic genomes with high rates of chromosomal rearrangements and probably low rates of synteny conservation, our results evidence that large syntenic chromosome segments have been maintained conserved during evolution, at least for the considered markers. Additionally, our current cytogenetic mapping efforts integrated with genomic approaches conduct to a new perspective to address important questions involving chromosome evolution in fishes.

## Background

Integrated genome maps became a powerful tool to fill the gaps generated by the low resolution of linkage mapping and the problems of genome sequencing and assembly, providing a more accurate scenario of the genome structure. The application of fluorescent *in situ* hybridization (FISH) based in the use of bacterial artificial chromosome (BAC) clones as probes represents an efficient approach to anchor genomic and linkage data on physical chromosomes. BAC libraries have been explored for many aspects of molecular and genomic studies, such as the positional cloning of genes [1], comparative studies of synteny and gene organization among different species [2], as well as for local or whole genome physical and genetic mapping and sequencing [3]. In cytogenetic research and chromosome mapping, the potential of BACs for animal genome analyses has improved, since several good quality genomes are already available in public databases [4] bringing up the possibility to refine the chromosome analysis under the focus of BAC-FISH mapping [5-12].

Cichlid fishes represent a monophyletic group classified in 4 subfamilies: Etroplinae (India and Madagascar), Ptychochrominae (Madagascar), Cichlinae (Neotropical region) and Pseudocrenilabrinae (Africa) [13,14]. Although African Pseudocrenilabrinae cichlids can be separated in three major groups (hemichromines, haplochromines, and tilapiines), these groups are not recognized as valid taxonomic units [15]. The African groups of lakes Victoria, Malawi and Tanganika represent a classical example of extensive and rapid radiation and therefore are highly interesting for evolutionary biology analyses [16]. The karyotypes of more than 135 cichlid species have been determined, evidencing a diploid chromosome number ranging from 2n = 32 to 2n = 60. African cichlids have a modal diploid chromosome number of 44 chromosomes whereas the Neotropical cichlids contain 2n = 48 chromosomes reviewed by [17].

Genetic maps containing a few hundred polymorphic markers provide a starting point in order to resolve the chromosomal location of cloned genes or markers. Genetic linkage maps have been developed for a number of fish species, including zebrafish [18], medaka [19], catfish [20], rainbow trout [21], Atlantic salmon [22,23], and also cichlids as the Nile tilapia *Oreochromis niloticus* (tilapiine) [24], Lake Malawi haplochromines [25], and *Astatotilapia burtoni*[26]. There are also other genetic/genomic resources for cichlids including extensive collections of expressed sequence tags (ESTs) for Lake Victoria haplochromines [27,28], *A. burtoni*[29,30], and *O. niloticus*[31], BAC libraries for Nile tilapia [32], and haplochromines from lakes Malawi [33], Victoria [34], and Tanganika [35], and a high-resolution map for Nile tilapia [36]. Altogether, these genomic resources have driven the investigation of several aspects of cichlid’s biology, including sex determination [24,37-39]. Tilapiines have an XY sex chromosome system on linkage group (LG) 1 or a ZW system on LG3 [7,40]. However, at least two distinct genetic sex determination systems in the Lake Malawi cichlids were found: a XY sex-determination system on LG7 and a ZW system on LG5 [41]. Furthermore, some genes observed in LG1, *CYP19a* and *WT1*, are involved in the sexual differentiation of mammals [42,43]; the *CLCN5* gene (associated with renal disorder in humans), located on LG3, turns to be interesting since it was detected in the human X chromosome [44]; the opsin genes responsible for the color-spectrum vision of cichlids and thus involved with sexual selection and adaptation to new environments are located in LG5 [45]. Additionally, the genome of *O. niloticus* has been recently sequenced [46], leading to the opportunity to integrate nucleotide sequence information and other genetic data. In this context, we address the comparative analysis of LG1, 3, 5, and 7 of cichlids based on the integration of genomic and molecular cytogenetic. Furthermore, the combined genomic/cytogenetic information obtained for cichlids was also comparatively analyzed with other vertebrates. Our results provide evidence for extensive synteny conservation of segments among Pseudocrenilabrinae cichlids and also between cichlids and other vertebrates. Such information is promising in the establishment of a framework for additional genome-wide studies.

## Results

### BAC-FISH mapping

**Figure 1:**
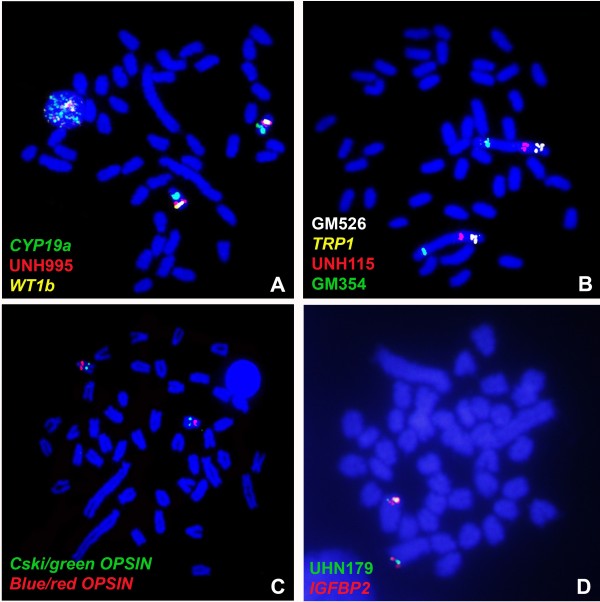
**BAC-FISH mapping of* O. niloticus *.** (**A**) Co-hybridization of three differentially labeled LG1 BAC-clones**.** (**B**) Co-hybridization of four differentially labeled LG3 clones to the largest chromosome. Dual-colour FISH of (**C**) LG5 and (**D**) LG7 markers. The chromosomes are counterstained with DAPI and the hybridized markers are indicated in different colors. Scale bar = 10μm.

**Figure 2:**
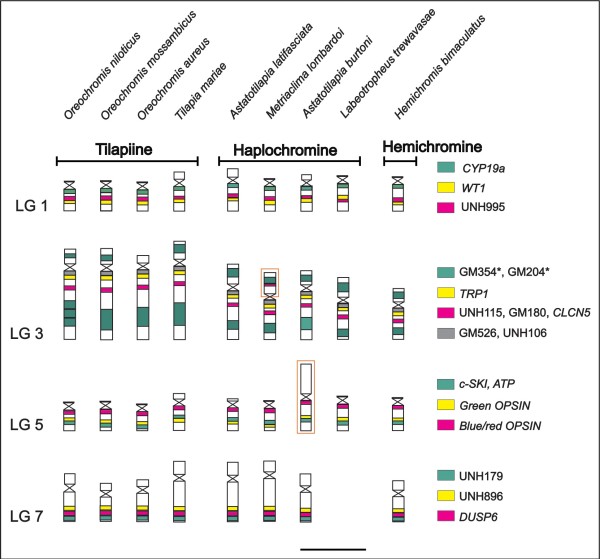
**Chromosomal homologies revealed by BAC-FISH.** The figure summarizes the results of the comparative FISH mapping of LG1, 3, 5, and 7 markers in nine Pseudocrenilabrinae species. For more details see Additional file 2. The different colors indicate the mapped markers (detailed on the left), and the orange bordering highlights the large metacentric chromosome carrying LG5 in *A. burtoni* and the duplicated marker (UNH115) in the LG3 of * M. lombardoi *. The asterisks (*) in the LG3 markers indicate the presence of repetitive DNA sequences. Linkage group 7 was not analyzed in * L. trewavasae*. Scale bar 5μm.

The chromosome diploid number and morphology were analyzed in Giemsa-stained metaphases (Table 1). Such analysis confirmed the previous karyotype data available for cichlids composed of meta and submetacentric (m/sm), and telo/acrocentric (t/a) chromosomes [17,47]. Metaphases of males and females of *O. niloticus* (the species source of the BAC clones used for FISH) were used to set up the BAC-FISH mapping experiments (Figure 1). After that, a comparative mapping, including eight other Pseudocrenilabrinae species, was carried out to elucidate the evolutionary history of chromosomes carrying LG1, 3, 5, and 7 (Figure 2, Additional file 1).

**Table 1:** **Cichlidae species submitted to cytogenetic mapping using BAC clones of* O. niloticus *as probes in FISH experiments**

**Subfamily**	**Groups or tribes**	**Species**	**2n**	**Sex**	**Karyotype**	**Origin**
Etroplinae		*Etroplus maculatus*	46	1M/1F/1ns	18m/sm+18t/a+10 micro	Petshop, Botucatu, Brazil
Pseudocrenilabrinae	Tilapiine	*Oreochromis niloticus*	44	2M/3F/1ns	2m/sm+42t/a	TAF-UMD
		*Oreochromis mossambicus*	44	1M/1F	4m/sm+40t/a	TAF-UMD
		*Oreochromis aureus*	44	1F	2m/sm+42t/a	TAF-UMD
		*Tilapia mariae*	40	1M/1F	8m/sm+32t/a	TAF-UMD
	Haplochromine	*Astatotilapia latifasciata*	44	2M/3F/1ns	12m/sm+32t/a	Petshop, Botucatu, Brazil
		*Astatotilapia burtoni*	40	1M/2F	14m/sm+26t/a	TAF-UMD
		*Metriaclima lombardoi*	44	1M/3F/1ns	14m/sm+30t/a	TAF-UMD
		*Labeotropheus trewavasae*	44	2M/3F	14m/sm+30t/a	TAF-UMD
	Hemichromine	*Hemichromis bimaculatus*	44	2 ns	4m/sm+40t/a	Petshop, Botucatu, Brazil
Cichlinae	Cichlini	*Cichla kelberi*	48	2M	48 t/a	Araguaia River, Brazil
	Astronotini	*Astronotus ocellatus*	48	2M	12m/sm+36t/a	Tietê River, Brazil
	Heroini	*Symphysodon aequifasciatus*	60	3ns	46m/sm+4t/a+10micro	Petshop, Botucatu, Brazil
	Geophagini	*Geophagus brasiliensis*	48	1M/1F	2m/sm+46t/a	São Paulo State rivers, Brazil

2n, diploid chromosome number; m/sm, metacentric/submetacentric; t/a, telocentric/acrocentric; micro, microchromosomes; M/F, males/females; ns, not identified sex.

Four BACs from LG1 mapped on the long arm of a small t/a chromosome pair of *O. niloticus* (Figure 1A). The largest chromosome of *O. niloticus* evidenced labeling signals of eight BACs of LG3 (Figure 1B) and the LG3 mapping confirmed the presence of a lot of repetitive DNA in the end of the largest chromosome of all analyzed species (Figure 2).

Three BACs from LG5 mapped on a medium t/a chromosome different from that containing the LG1 (Figure 1C), and three BACs from LG7 mapped on the second largest pair of *O. niloticus* (Figures 1D). No differences were found between males and females for any of the hybridized BAC-probes.

At least two individuals of each species were used in BAC-FISH experiments and the signal patterns observed for LG1, 3, 5, and 7 were conserved in number and position when compared to the reference species *O. niloticus* (Figure 2; See Additional file 1). No results were obtained for BACs of LG7 in *Labeotropheus trewavasae* due to the low quality chromosome preparations of this species for FISH procedures (Figure 2). In South American cichlids (Cichlinae), none of the BAC probes produced identifiable chromosomal signals. Furthermore, no hybridization signal was observed in the Asian Etroplinae species, *Etroplus maculatus*.

Besides the conservation of the studied LGs, some differences were observed in the chromosomes’ morphology of the species (Figure 2): *Astatotilapia burtoni* showed a different pattern for LG5 which is located on the short arm of a large m/sm chromosome instead of a smal t/a chromosome as observed in the other cichlids. The single BAC signal observed in the short arms of the chromosomes was related to repeated sequences as observed for the UNH115 marker in LG3 of *Metriaclima lombardoi* (Figure 2; Additional file 1). Additionally, small variations in the chromosomal position of the markers were also observed (See Additional file 1), but these could be just a consequence of variation in the chromosome condensation of the analyzed individuals.

**Figure 3:**
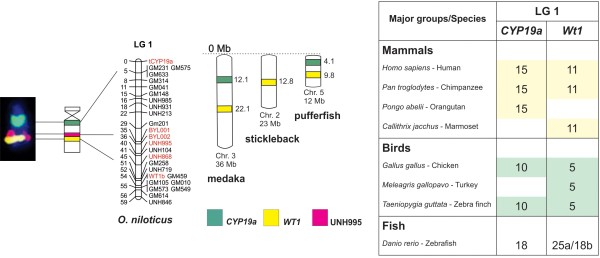
**Comparative analyzes of LG1 showing the conservation of genomic blocks among Nile tilapia, medaka, stickleback, pufferfish, zebrafish, and other vertebrates.** BAC-labeled metaphase chromosomes are showed on the left, followed by ideograms and LG1 of *O. niloticus*. The three markers (* CYP19a *, * WT1 *, and UNH995) are identified by different colors and the chromosome positions for medaka, stickleback, and pufferfish are indicated in Mb on the right of the idiogram. The table on the right summarizes the results of comparative analyzes with other vertebrates. The numbers represent the identified chromosome and the conserved genomic regions are highlighted in different colors. The position of the markers in the chromosomes can be found at www.ensembl.org.

**Figure 4:**
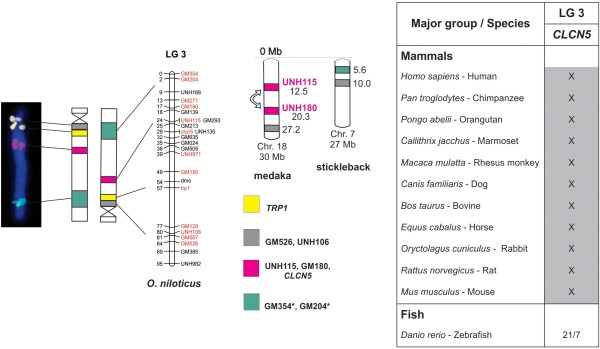
**Comparative analyzes of LG3 showing the conservation of genomic blocks among Nile tilapia, medaka, stickleback, zebrafish, and other vertebrates.** BAC-labeled metaphase chromosomes are shown on the left, followed by ideograms and LG3 of *O. niloticus*. The ideogram was inverted according to LG3 orientation. The markers (*TRP1, CLCN5*, UNH115, GM180, GM526, UNH106, GM354, and GM204) are identified by different colors and the chromosome positions for medaka and stickleback are indicated in Mb on the right of the idiograms. The table (on the right) summarizes the results of comparative analyzes with other vertebrates. The numbers represent the identified chromosome and the conserved genomic regions are highlighted in different colors. The position of the markers in the chromosomes can be found at www.ensembl.org.

**Figure 5:**
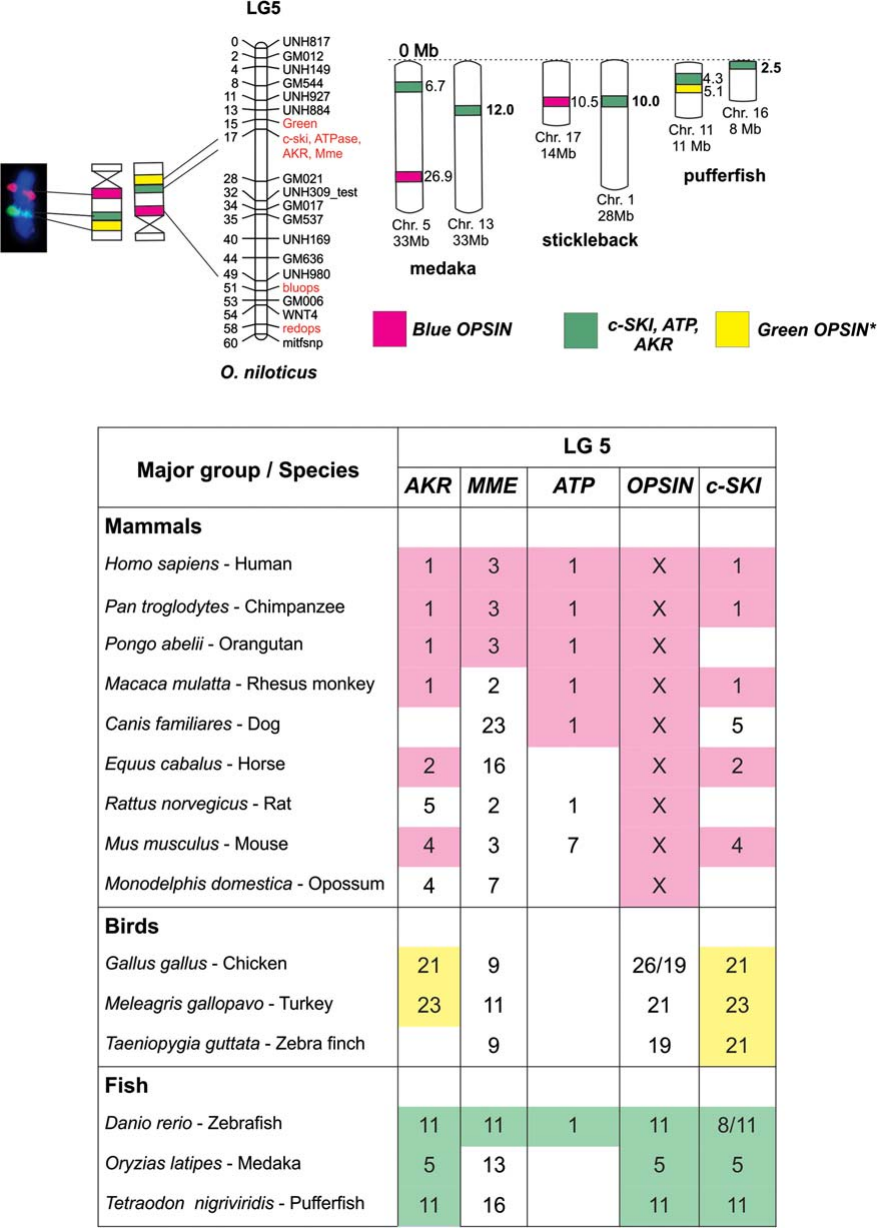
**Comparative analyzes of LG5 showing the conservation of genomic blocks among Nile tilapia, medaka, stickleback, pufferfish, zebrafish, and other vertebrates.** BAC-labeled metaphase chromosomes are showed on the left, followed by ideograms and LG5 of *O. niloticus*. The ideogram was inverted according to LG5 orientation. The four markers (*Blue/red OPSIN, Green OPSIN, c-SKI,* and *ATP*) are identified by different colors and the chromosome positions for medaka, stickleback, and pufferfish are indicated in Mb on the right. The table (below the comparative maps) summarizes the results of comparative analyzes with other vertebrates. The numbers represent the identified chromosomes and the conserved genomic regions are highlighted in different colors. The position of the markers in the chromosomes can be found at www.ensembl.org.

**Figure 6:**
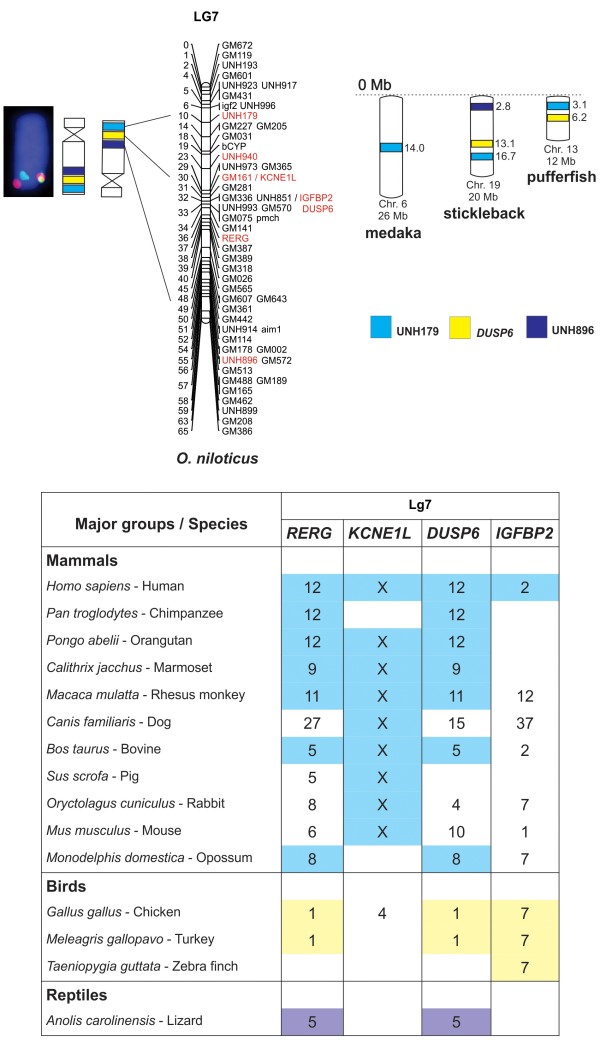
**Comparative analyzes of LG7 showing the conservation of genomic blocks among Nile tilapia, medaka, stickleback, pufferfish, and other vertebrates.** BAC-labeled metaphase chromosomes are showed on the left, followed by ideograms and LG7 of *O. niloticus*. The ideogram was inverted according to LG7 orientation. The three markers (UNH179, *DUSP6*, and UNH896) are identified by different colors and the chromosome positions for medaka, stickleback, and pufferfish are indicated in Mb on the right. The table (below the comparative maps) summarizes the results of comparative analyzes with other vertebrates. The numbers represent the chromosomes identified and the conserved genomic regions are highlighted in different colors. The positions of the markers in the chromosomes can be found at www.ensembl.org.

The chromosomal positions of all selected BACs were correlated with their corresponding order on their linkage maps. This enabled the association of LGs with chromosomes and to orientate their position according to the short/long arm of the corresponding chromosomes. Using the convention that the short arm is north oriented to and the long arm is south oriented, the north/south orientation of the current *Oreochromis* LG1 matches with the short/long arm (Figure 3); however, the orientation of LG3, 5, and 7 should be inverted in relation to the order of previously published molecular markers (Figures 4, 5 and 6).

### In silico comparative analyses

Comparative genomic analyzes of LG1 at Bouillabase genome browser suggested a conserved pattern of these markers in medaka chromosome 3, stickleback chromosome 2, and pufferfish chromosome 5 (Figure 3) (Table 2). Additionally, using Ensembl and Genomicus databases, the chromosomal position of the genes *WT1b* and *CYP19A1*, mapped in LG1, was comparatively analyzed among fishes and also other vertebrates (Figure 3). The present analyses showed that *WT1b* and *CYP19A1* genes are located at chromosome 18 of *Danio rerio* (zebrafish) (Figure 3) and separated by 6Mb. In medaka and pufferfish, these genes are separated by 10Mb and 5Mb, respectively (Table 2). A divergent scenario occur in other vertebrates, in which these genes appear in different chromosomes as observed in chicken and primates, where both markers are in chromosomes 10 and 5, and chromosomes 15 and 11, respectively (Figure 3).

**Table 2:** **BouillaBase comparative analyzes using BAC data from* Oreochromis niloticus *against three fish models**

**LG**	**Marker/gene**	**Scaffold**	**Medaka**	**Stickleback**	**Pufferfish**
1	*CYP19A1*	287	chr3:12108168..12108464	ns	chr5:9794344..9794562
1	ACG/CTT382	17	chr3:20997422..20997535	chr2:12085900..12086028	chr5:4855030..4855202
1	BJ690985	NID	chr19:4811402..4811495	ns	ns
1	*WT1*	NID	chr3:22126505..22127094	chr2:12884672..12885323	chr5:4189140..4189790
3	UNH180	143	chr18:20335074..20335221	Ns	chrUn:92504595..92504738
3	UNH115	NID	chr18:12554314..12554397	chrUn:7877924..7878007	chrUn:98704287..98704370
3	*TRP1*	88	chrUn:35101810..35101948	chr7:5614225..5614569	chrUn:56261613..56261704
3	GM526	89	chr18:27239660..27426308	chr7:10064534..10064979	ns
5	*AKR*	155	chr5:7748231..7748360	chrUn:4859687..4859812	chr11:3868317..3868442
5	*AKR*	155	chr5:7708974..7709058	chrUn:4889317..4982133	chr11:3884393..3884499
5	*MME*	340	chr13:11994389..11995120	chr1:10620278..10620987	chr16:2528394..2528542
5	*MME*	162	chr13:12106428..12106606	chr1:10575763..10575935	ns
5	*Green OPSIN*	NID	chrUn:142344721..142344800	chrUn:4181024..4181104	chr11:5142370..5142445
5	*Blue/red OPSIN*	19	chr5:26959747..26959838	chr17:10579335..10579447	ns
5	*c-SKI*	NID	chr5:6600759..6600879	chrUn:3593246..3593357	chr11:4411020..4411130
5	*c-SKI*	64	chr5:6741965..6742076	chrUn:3505497..3505978	chr11:4336173..4336280
7	*DUSP6*	52	Ns	chr19:13,105,072..13,105,575	chr13:6,253,123..6,253,497
7	*IGFBP2*	NID	chrUn:75,506,034..75,506,204	chrUn:62,550,041..62,550,211	chr13:6,057,596..6,145,055
7	UNH179	NID	chr6:14,100,033..14,100,152	chr19:16,793,012..16,793,135	chr13:3,161,846..3,161,931
7	UNH896	NID	Ns	chr19:2,835,715..2,992,804	ns

NID, not identified scaffold in *O. niloticus*; ns, no similarity; chr, chromosome; chrUn, unidentified chromosome.

LG3 proved to be conserved with at least two correspondent markers between the fish species medaka (chromosome 18) and stickleback (chromosome 7). In the pufferfish genome, markers of LG3 mapped at a non identified region (Table 2). Despite the synteny of LG3 markers identified in medaka and stickleback, an inversion of the markers UNH115 and UNH180, separeted by 8 Mb, was detected in medaka in relation to *Oreochromis* (Figure 4).

LG3 contains few known genes that could be used for comparative analyses in other vertebrates. *CLCN5* was the only gene identified in other vertebrates. Using *CLCN5* in a comparative analysis against the zebrafish genome, this gene appears duplicated and located at chromosomes 7 and 21. On the other hand, in all mammal species, *CLCN5* has been conserved in X chromosome (Figure 4).

The majority of LG5 markers corresponds to chromosome 5 in medaka, chromosome 17 in stickleback, and chromosome 11 in pufferfish. Although other LG5 markers (*Green OPSIN* and *c-Ski*) were found in stickleback and medaka genome, it was not possible to categorize their chromosome position (Table 2). The position of the *MME* gene differs, as it can appear in chromosome 13, 1, or 16, respectively, in the three mentioned species (Table 2) (Figure 5). With the addition of zebrafish in Ensembl datasheet, an extremely conserved pattern of LG5 in chromosome 11 was evidenced. Once more, a duplicated marker was found in zebrafish (see *c-Ski*, for instance, in Figure 5). Genes observed in LG5 (*AKR, ATP* and *c-Ski*) have their positions conserved in chromosome 1 of primates. Additionally, this conservation is also related to horse chromosome 2, mouse chromosome 4, chicken chromosome 21, and turkey chromosome 23 (Figure 5). Despite located in the same chromosome, the position of these genes are inconsistent - in *O. niloticus* these genes appear tight together at 17 cM; however, in other species they appear separated, sometimes around 70Mb apart (data not show). The *OPN* gene (*Blue opsin*), present in the end of *O. niloticus* LG5, matched in the X chromosome for all analyzed mammals (Figure 5).

Analysis of LG7 markers in BouillaBase showed a block of synteny between *Oreochromis*, stickleback chromosome 19 and pufferfish chromosome 13, even taking into account that the distances between these markers are similar among the species. In medaka genome, only the *UNH179* marker was identified in chromosome 6 (Figure 6). It was impossible to identify the position of the *IGFBP2* marker in medaka and stickleback genomes, but in pufferfish it is located together with *DUSP6* (Table 2), as also observed for *O. niloticus* (Table 2). In Ensembl comparative studies, the analysis of a small region (about 3cM) of LG7 that contains the genes *RERG*, *KCNE1L*, *DUSP6*, and *IGFBP2*, revealed a conserved position of *RERG* and *DUSP6* at the same chromosome in vertebrates: *Homo sapiens* chromosome 12, *Macaca mulatta* chromosome 11, *Bos taurus* chromosome 5, *Gallus gallus* and *Meleagris gallopavo* chromosome 1, and indeed in the reptile *Anolis carolinensis* chromosome 5. However, these genes are separated by 10 to 75 Mb (data not shown) in non-fish species (Figure 6). Moreover, the gene *KCNE1L* was conserved in the X chromosome of all mammals (Figure 6).

## Discussion

### Cytogenetic mapping and chromosome stability in Pseudocrenilabrinae

The most important find in our study is the extreme chromosome conservation observed within African cichlids. It is already known that African cichlids (Pseudocrenilabrinae) comprise about 1.400 species [48] and that their modal chromosome number is 2n = 44 [17]. Extensive comparative mapping has demonstrated that the genetic maps for tilapia and Malawi cichlids are almost perfectly collinear [41]. This conservation could be evidenced through comparative physical chromosomal mapping of linkage groups as shown in the present study. On the other hand, variability at the cytogenetic level among cichlids was mostly observed based in repetitive DNA chromosome mapping [49,50] and seems to reflect the evolutionary dynamics of the repetitive genomic fraction and not broad processes acting in the whole karyotype.

Within Pseudocrenilabrinae, the major karyotype feature in the tilapiines is the presence of one large t/a chromosome pair (LG3), which is significantly larger than all other elements of the karyotype [17,51]. On the other hand, the haplochromine and hemichromine cichlids have shown two outstanding chromosome pairs [17,52] - the first and the second largest elements that correspond to LG3 and LG7, respectively (Figure 2). The chromosome mapping of LG3 and LG7 markers (present report) in Pseudocrenilabrinae species confirms the proposed homology between the two largest chromosome pairs of tilapiines and haplochromines/hemichromines [52]. However, the hypothesis concerning an independent chromosome fusion that originated the largest chromosomes in tilapiines and haplochromines [52] is not in agreement with the results observed for the cytogenetic mapping of LG3 and LG7 markers.

It is known that the largest chromosome pair of *O. niloticus* originated by a centric fusion event of three other pairs of the ancestral cichlid karyotype composed of 48 acrocentric chromosomes [53]. A previous study [52] proposed that a first chromosome fusion took place before the divergence of the main East African cichlid groups. The second chromosome fusion occurred independently in the tilapiines and non-tilapiines. In the tilapiines, a new chromosome was fused to the largest pair, and in the non-tilapiines, the second fusion did not involve the largest chromosome, but two other chromosomes, which gave rise to the chromosome pair 2. However, when we compared the distribution of BAC signals (LG3 and LG7) through the long arm of the two largest chromosomes in tilapiines and non-tilapiines species, we detected that the second chromosome fusion seems to be identical in these two groups, producing a chromosome with the same genomic content that remained conserved in all Pseudocrenilabrinae species so far analyzed (Figure 2).

The differences observed in the chromosome size of the first pair between tilapiines and haplochromines could be due to the intense dynamics of repeated DNAs located in the entire long arm instead of differences related to possible chromosome fusions. A range of studies has shown that the largest chromosome in *O. niloticus* presents a great amount of heterochromatin, microsatellites, transposable elements, LINES and SINES, and non-LTR retrotransposons [52-59]. The positive results using LG3-BACs (enriched of repetitive DNAs, see Table 3) evidenced the accumulation of repeated DNAs in the LG3 chromosome of all Pseudocrenilabrinae, specially in tilapiine species.

**Table 3:** Genetic markers and their BAC identification (ID)

**Markers**	**LG**	**BAC 384 Well ID**	**Gene**
UNH995	LG1^+^	b04TI071H11	
	LG1	b04TI008J05	*CYP19A*
	LG1	b04TI002B08	*CYP19A*
	LG1	b03TI091I08	*WT1*
GM354	LG3^+^	b03TI066P02	
GM204	LG3^+^	b04TI071O04	
UNH180	LG3^+^	b04TI056G07	
UNH115	LG3^+^	b03TI086K09	
	LG3^+^	b04TI076F11	*CLCN5*
	LG3	b03TI073M01	*TRP1*
UNH106	LG3	b03TI088C14	
GM526	LG3	b03TI067N14	
	LG5	b04TI053F24	*ATP*
	LG5^+^	b04TI010O22	*Green OPSIN*
	LG5	b04TI075I09	*Blue OPSIN*
	LG5	b04TI006L21	*c-SKI*
	LG7	b03TI050E01	*RERG*
	LG7^+^	b03TI079D23	*KCNE1L*
	LG7	b03TI080A15	*DUSP6*
	LG7	b03TI081O07	*IGFBP2*
UNH179	LG7	b04TI036P14	
UNH896	LG7	b04TI035B08	

^+^: Presence of repetitive DNAs.

Despite displaying a highly conserved karyotype structure, many events of duplication, inversion, and fusion occurred during the diversification of African cichlids and resulted in diploid chromosome numbers divergent from the pattern of 44 chromosomes, as observed in *Tilapia mariae* with 2n = 40 [52,60], *T. sparrmanii* with 2n = 42 [60], *Oreochromis alcalicus* with 2n = 48 [17], *O. karongae* with 2n = 38 [10], and *Astatotilapia burtoni* with 2n = 40 [17], among others.

*A. burtoni* has two metacentric chromosome pairs, which are probably the result of centric fusions of two small t/a chromosome pairs. According to our results, the ancestral t/a chromosome that contains the LG5 (observed in other haplochromines) might be involved in one of these events resulting in a m/sm chromosome. Such assumption could not be inferred for *T. mariae* (tilapiine), in which the diploid chromosome number reduction to 2n = 40 must be the result of rearrangements that did not involve the linkage groups investigated here (Figure 2).

Additionally, the reduction of chromosome number observed in *O. karongae* (2n = 38) and the presence of three pairs of medium- sized chromosomes, that are not found in the typical *Oreochromis* species, were originated by chromosome fusion events involving LG1 [10,61] and could represent recent chromosomal rearrangements that have occurred independently in tilapiine and haplochromine groups. The additional signal observed in the 1p arm (LG3 – *UNH115*) of the largest chromosome of males and females of *M. lombardoi* could be a consequence of specific rearrangements or, even more, the presence of chimeric BAC inserts.

The absence of BAC-FISH signals in South American cichlids (Cichlinae) belonging to different tribes (Table 1) could be associated to genomic rearrangements that have disrupted in a small-scale level the genomic blocks carried by the BAC clones of *O. niloticus* in relation to these cichlines. Instead of being a monophyletic group [13], Neotropical cichlids harbor significantly higher levels of genetic variation compared to the African Pseudocrenilabrinae group [62]. Although genomic rearrangements seem to have occurred differentiating South American and African cichlids, it is plausible that large genomic blocks are still conserved between them as well as in relation to other fish groups. Unfortunately, deep analyses integrating cytogenetics and genomic data were not possible for Cichlinae species since there is no available large-scale genome information for this subfamily so far.

### Comparative analyses of vertebrates

Using BAC-end sequence data available at BouillaBase, it was possible to perform comparative analyses and detect regions of synteny among African cichlids and model fish species, and also vertebrates. In this work, conserved (syntenic) chromosome segments have been successfully identified by means of comparative cytogenetics between cichlid markers (mainly LG5 and 7) and mammals and birds chromosomes. The marker distances are very similar among the analyzed teleosts. However, considering non-fish groups, the markers’ distances are, in most cases, very divergent, evidencing that genomes are suffering rearrangements, despite the maintenance of conserved large genomic blocks as part of the same LG. Although large genomic blocks are conserved among vertebrates, variability in the genes/DNA sequences harbored in these regions is expected.

Comparative BAC mapping has already shown that some stickleback chromosomes have a nearly complete synteny with those of cichlids. Although stickleback does not belong to the order Perciformes, it is considered to be the closest related genome to cichlids among the currently available sequenced teleost genomes [63] and is currently the best reference sequence for assembling comparative maps of tilapia [64].

A range of studies using comparative cytogenetics has demonstrated that karyotypes are conserved in a macro scale throughout vertebrates. In Canidae, for example, despite the extensive variation in chromosome numbers and morphology, the majority of conserved chromosome segments appear to have remained largely intact in the karyotypes of extant canid species, although the relative orientation and distances are not always conserved [65]. Conserved karyotypes are also observed in birds, which show a slow rate of interchromosomal rearrangements [9], and within the reptile family Scincidae, a character that was shown by cross-species chromosome painting [66]. Similarly, at different taxonomic levels, comparative gene mapping has revealed a highly conserved linkage homology between an agamid lizard (*Leiolepis reevesii*) and a snake (*Elaphe quadrivirgata*) [67].

Conservation of large genomic blocks were also identified among different species of the Salmonidae family based on BAC-FISH blocks [8], and on *SOX* genes regions in cichlids that revealed a large genomic block that was conserved through vertebrates [12]. These conservations of gene orders at scales of several Mb in diverse vertebrate groups permit the use of relatively complete sequences of model fish species to accelerate gene discovery and positional cloning of non-model species [63,68]. The synteny observed in different vertebrates could be due to intrinsic chromosomal properties that confer selective pressure on large genomic blocks that should be preserved from major changes [69].

There is a classical idea that fish genomes have high rates of chromosomal rearrangements compared to other vertebrates and then, probably, evidence low rates of synteny [70-73]. However, our data associated to previous genomic studies are revealing that when we compare the genomic synteny among teleosts and other vertebrates in a macro scale level, large syntenic blocks can be clearly identified.

### Comparative analysis of sex chromosome regions

Three genes from different LGs of cichlids (*CLCN5*-LG3*, OPSIN*-LG5, and *KCNE1L*-LG7) are located on the X chromosome of human and other mammals, highlighting the conservation of this sex chromosome through mammals [74]. However, in bird clade these genes do not correspond to the Z or W chromosomes and, instead of this, are located in autosomes. This is explained by the fact that the ZW and XY chromosomes have no homology and both sexual chromosome pair systems were derived from different autosomes from their common ancestors [75].

In contrast to mammals and birds, the pufferfish, like most fish, does not possess heteromorphic sex chromosomes, and the genetic mechanism(s) of sex determination is still unclear. However, the human X is a mosaic of orthologs from three chromosomes of the pufferfish: most human Xp and Xq genes are syntenic on pufferfish chromosomes 1, 2, and 7 [69]. In addition, zebrafish LGs 9 and 23 are also related with both human Xp and Xq orthologs [18,76].

The largest chromosome of Nile tilapia that contains the LG3 was previously thought to be the sex chromosome of this species [77-80]. However, the major sex-determining region in the Nile tilapia was mapped on LG1 [81] located in a small t/a chromosome [present work, 7]. LG1 also contains the *WT1* and *CYP19a* genes involved in mammalian sex differentiation [42], although they are not considered anymore to be candidate genes for sex determination in African cichlids [38]. Even though our results are still limited, the integrated comparative analysis approach seems to be promising in the clarification of the complex evolutionary dynamics of sex chromosomes among fishes.

## Conclusions

Although African cichlids present karyotype variations related to number and morphology of the chromosomes, the linkage groups that were currently investigated (LG1, 3, 5, and 7) were preserved from major changes during their chromosomal diversification. The linkage of large chromosome blocks among cichlids was also preserved in other fish and vertebrates.

The use of BACs containing genes/markers represents a promising alternative for a better physical mapping for cichlids, and the integration of BAC-FISH maps to genomic data stands for a powerful tool to support a better assembling of genomes, contributing in the establishment of a framework for comparative genome-wide studies.

## Methods

### Animals and sampling

Cichlids from Lake Malawi were collected from the wild from 2005–2008 and maintained in the Tropical Aquaculture Facility (TAF) of the University of Maryland (UMD), College Park, MD, USA. Additional African and Asian species of uncertain origin were obtained from commercial sources in Botucatu, SP, Brazil, and South American species were collected from the wild in Brazilian rivers (Table 1) and maintained in the fish room of the Laboratory of Genômica Integrativa (FR-LGI) at Sao Paulo State University (UNESP), Botucatu, SP, Brazil. All the examined specimens were fixed in formaldehyde and then stored in alcohol in the fish collections of TAF-UMD and FR-LGI.

### BAC clones and probes labeling

BAC clones containing specific markers of LG1, 3, 5, and 7 (Table 3) were obtained from a BAC library of the Nile tilapia, *O. niloticus*, which was previously developed [32], and were used as probes for FISH. BAC extraction was conducted using the PhasePrep®^TM^ BAC DNA Kit (Sigma-Aldrich, St Louis, MO, USA) according to supplier’s protocol. The BAC clones were labeled with biotin, digoxigenin coupled nucleotides (Roche Applied Sciences, Indianapolis, IN, USA), CY3- and CY5-avidin (GE-Healthcare, UK) using whole genome amplification (WGA2 & 3 kits) (Sigma-Aldrich), according to the supplier’s protocol. After that, DNase-I (Sigma-Aldrich) concentration was titrated to yield labeled DNA fragments ranging from 100 to 500 base pair products. For multicolor FISH, we used 16 μl of probe mixture containing: 10 μl of hybridization mixture (62.5% deionized formamide, 12.5% of 20XSSC, 12.5% of dextran sulfate 50%), 4 μg of blocking DNA (salmon/herring sperm DNA or Cot-1 DNA resuspended in the hybridization mixture) and 100 nanograms of each probe. The probe mixture was denatured for 10 min at 65°C and immediately cooled on ice.

### Chromosome preparation and FISH procedure

Mitotic chromosomes of cichlid species belonging to the Pseudocrenilabrinae (including representatives of tilapiine, haplochromine, and hemichromine groups), Cichlinae and Etroplinae subfamilies (Table 1) were prepared from anterior kidney cells with *in vivo* colchicine treatment [82]. The slides with chromosomes were air-dried, treated with pepsin (0.01% in 10 mM HCl) and dehydrated in an ethanol series one day before use. The slides were denatured in 70% formamide/2xSSC, pH 7.0 for 40 s, and dehydrated in an ice-cold ethanol series. The probe mixture was hybridized under a 24 x 50 mm cover slip in a 37°C moist chamber for 48 h. Slides were washed two times for 5 min each in 50% formamide/2xSSC, pH 7.0 at 43°C under agitation, then 10 min in 2xSSC, pH 7.0 at 42°C under continuous agitation. For undirected labeled probes, the hybridization signals were detected with avidin-FITC and rhodamine-anti-DIG (Roche Applied Sciences, Indianapolis, IN, USA), according to the supplier’s protocol. After three washes of 2 min in phosphate buffer detergent (4xSSC/1% Tween-20), slides were mounted with antifade solution containing DAPI (Vectashield mounting medium). Results were recorded with an Olympus BX61 microscope equipped with an Olympus digital camera DP71 and the software Image-Pro MC 6.0.

The selected BACs were checked in order to identify if the FISH mapping results were in agreement to the anchoring marker positions on LGs. Each group of BACs from a particular linkage group was checked to ensure if it hybridizes to the same chromosome using at least two markers from each LG.

### Comparative genomic database analyses

The comparative analyses among *O. niloticus* BAC-end sequences and *Oryzias latipes* (medaka), *Tetraodon nigroviridis* (pufferfish) and *Gasterosteus aculeatus* (stickleback) were done based on the BAC ID (Table 3) of the clones used for FISH against the updated version of the comparative genome browsers of the BouillaBase database [46]. The BAC ID was the starting point to detect a landmark or region of similarity in the databank source. The identification of genomic positions of genes located in LG1, 3, 5, and 7 of *O. niloticus* in other vertebrates was determined using the currently available public genomic databases Sanger Institute Ensembl Database [83], Genomicus genome browser [84], and NCBI Map Viewer [85]. Once a query gene name has been entered in the search box, the position of the gene in all species that have their genome available and annotated was retrieved. The accession numbers of all genes used in the comparative analyze are available as Additional file 2.

## Abbreviations

AKR, Aldo-keto reductase; ATP, ATPase, Na+/K + transporting; BAC, Bacterial artificial chromosomes; CLCN5, Chloride channel 5; c-Ski, v-ski sarcoma viral oncogene homolog; CYP19A1, Cytochrome P450, family 19, subfamily A, polypeptide 1; DUSP6, Dual specificity phosphatase 6; ESTs, Expressed sequence tags; FISH, Fluorescence in situ hybridization; IGFBP2, Insulin-like growth factor binding protein 2; KCNE1L, Potassium voltage-gated channel, Isk-related family, member 1-like; LG, Linkage groups; m/sm, Meta/submetacentric; MME, Membrane metallo-endopeptidase; OPN, Opsin – cone pigments; RERG, RAS-like, estrogen-regulated, growth inhibitor; SOX, Sex determining region Y; t/a, Telo/acrocentric; WT1b, Wilms tumor 1.

## Competing interests

The authors declare that they have no competing interests.

## Author’s contributions

JM carried out chromosome preparations, molecular cytogenetics experiments with BAC probes, *in silico* analyses and data interpretation , and drafted the manuscript. FY conceived the initial chromosome hybridization experiments with BAC probes, analyses and data interpretation and revised the manuscript. TDK helped in the analyses and data interpretation and revised the manuscript. CM conceived the study, participated in its design and coordination and helped to draft the manuscript. All authors read and approved the final manuscript.

## Supplementary Material

Additional file 1 **Metaphase spreads of cichlid species probed with BAC clones from*Oreochromis niloticus.*** BACs containing markers of LG1, 3, 5, and 7 were hybridized through FISH procedure and are indicated in different colors in each metaphase. The arrows indicate the chromosome position of probes. Click here for file

Additional file 2 **Genomicus/Ensembl accession numbers.** Datasheet with accession numbers and markers of all analyzed sequences in Genomicus/Ensembl database for the LG1, 3, 5, and 7. Analyzes were conducted in July 2011. Click here for file
