# Supplementary material for: Integrating cytogenetics and genomics in comparative evolutionary studies of cichlid fish
Source: BMC Genomics. 2012 Sep 9;13:463. doi: 10.1186/1471-2164-13-463 (PMC3463429; doi:10.1186/1471-2164-13-463)

Additional file 1: Metaphase spreads of cichlid species probed with BAC clones from *Oreochromis niloticus*. BACs containing markers of LG 1, 3, 5 and 7 were hybridized under FISH procedure and are indicated in different colors in each metaphase. The arrows indicate the chromosome position of probes. Scale bar 10µm.

Oreochromis niloticus

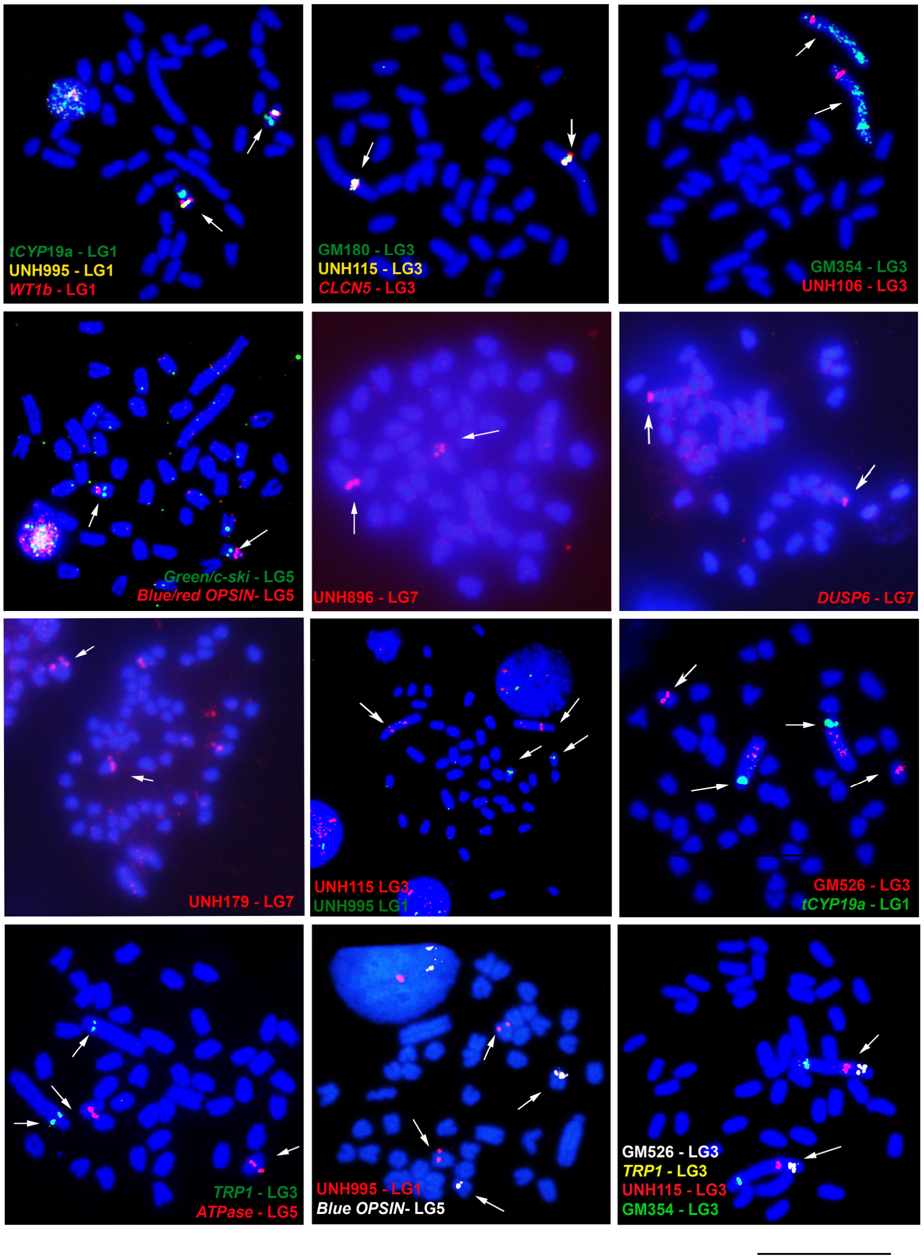

Oreochromis mossambicus

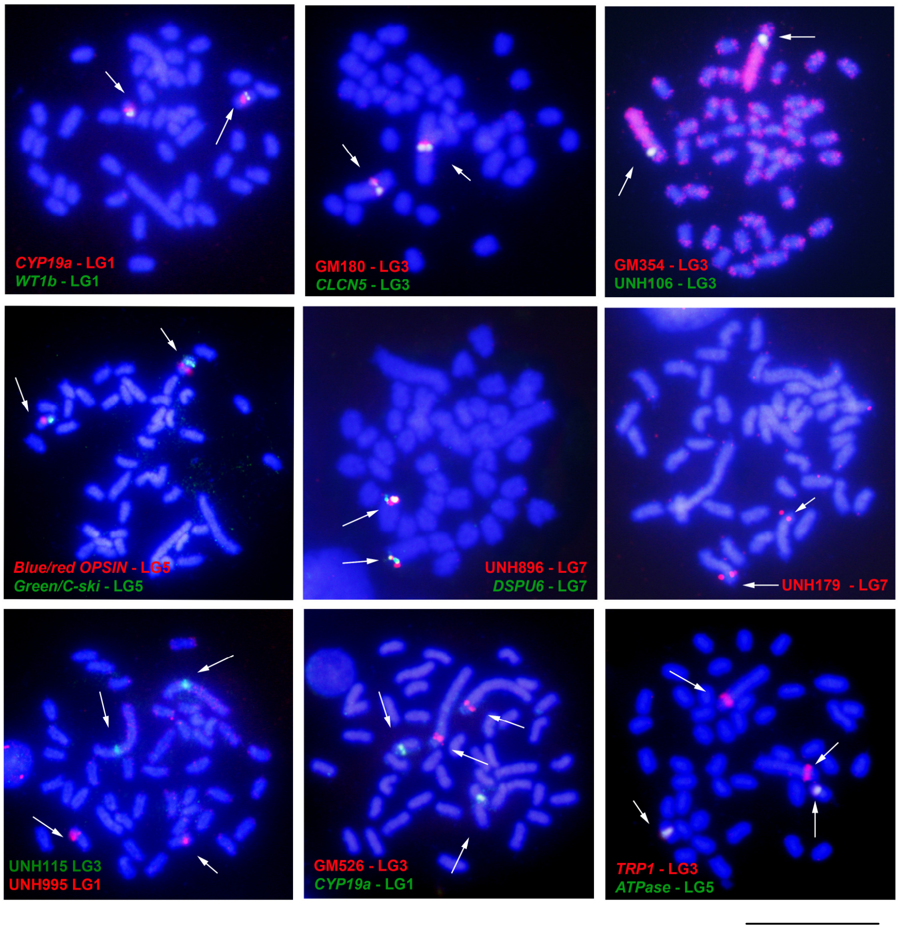

Oreochromis aureus

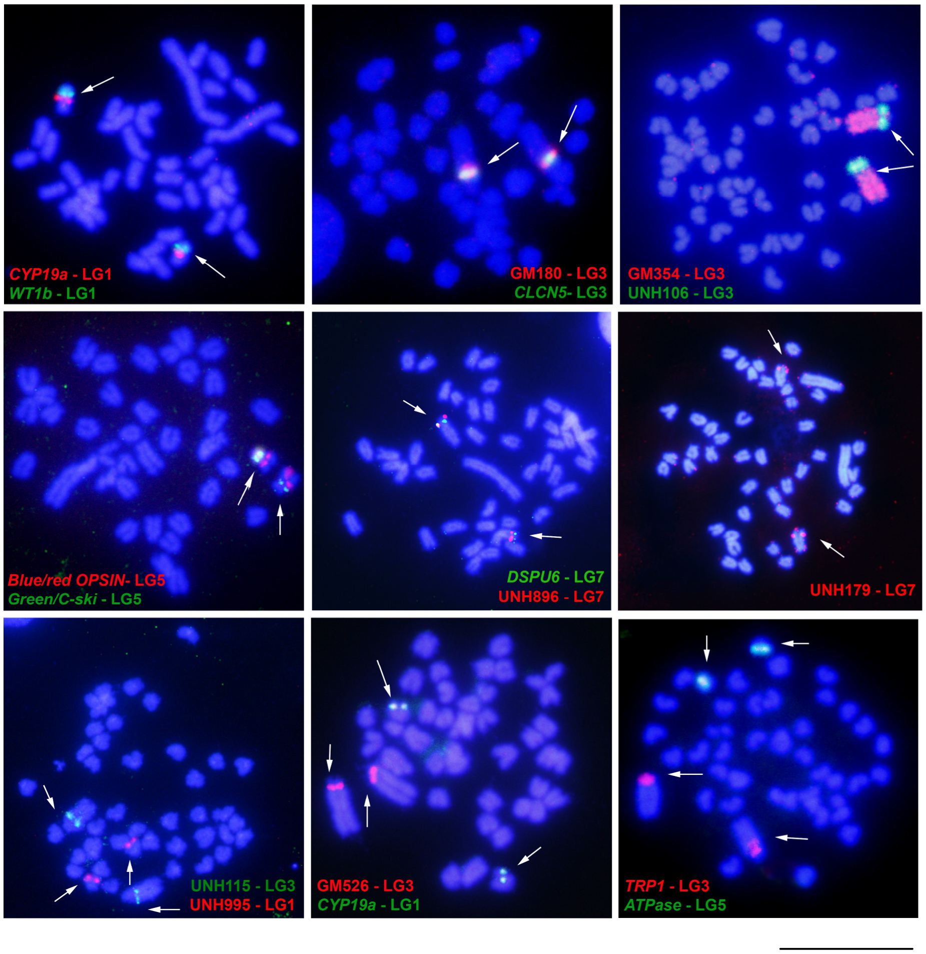

Tilapia mariae

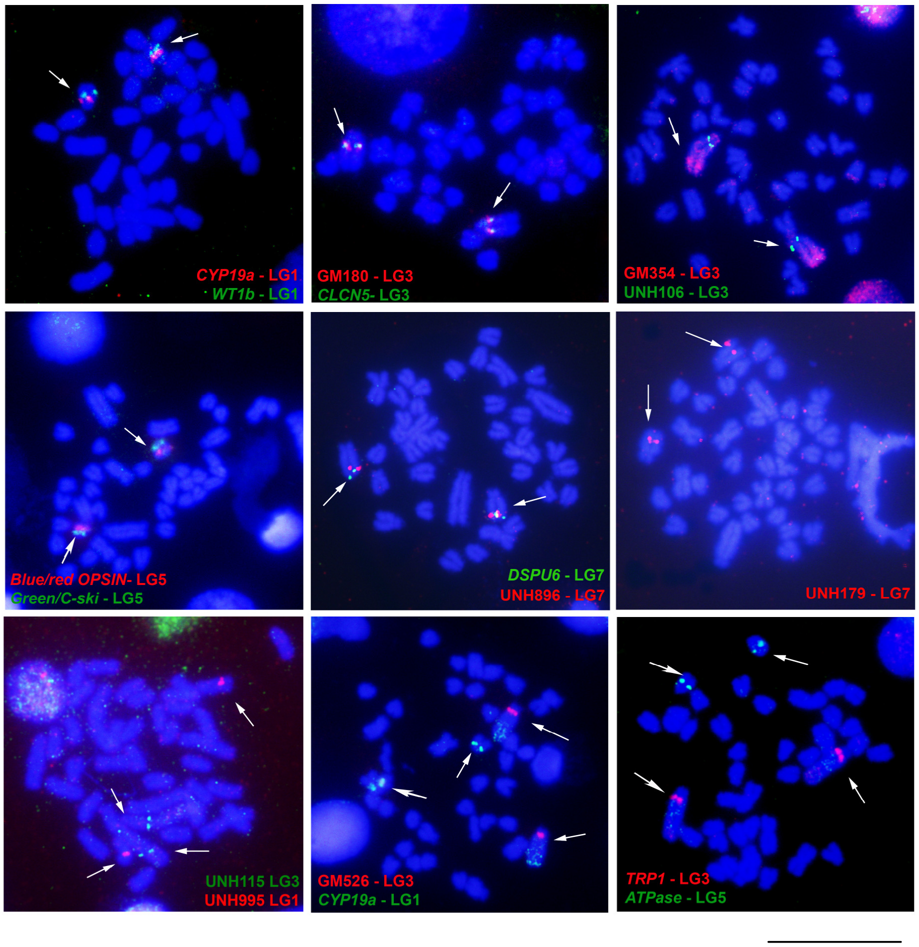

Astatotilapia latifasciata

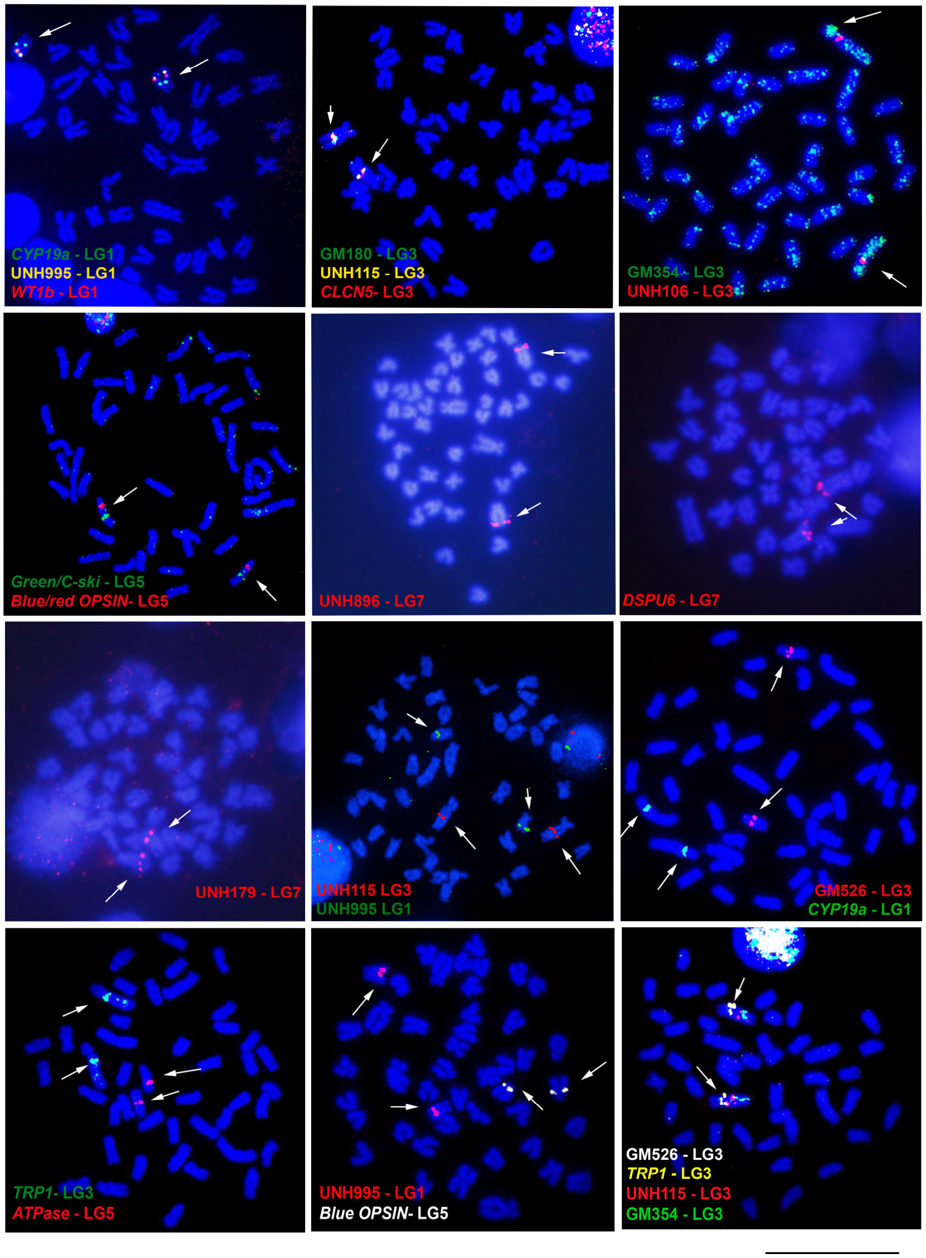

Metriaclima lombardoi

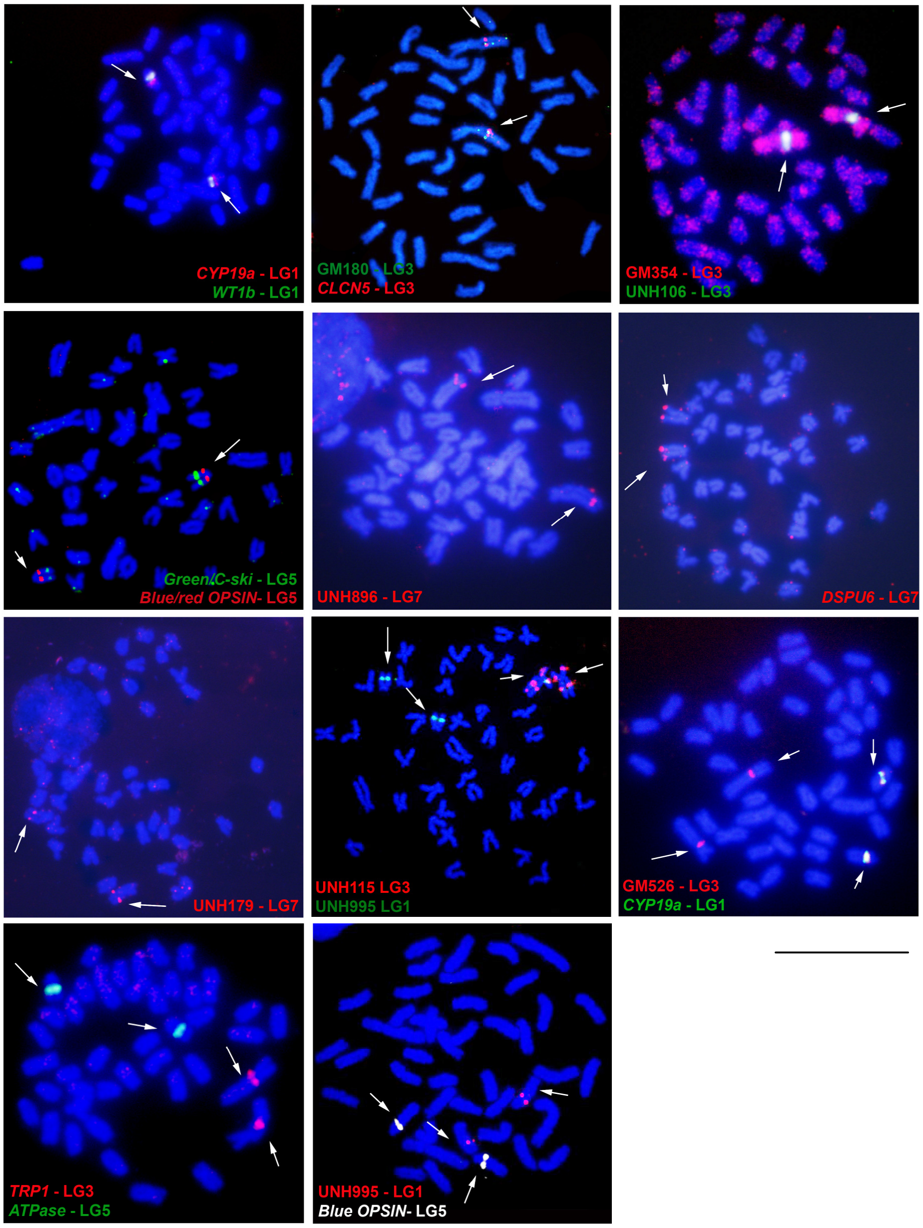

Astatotilapia burtoni

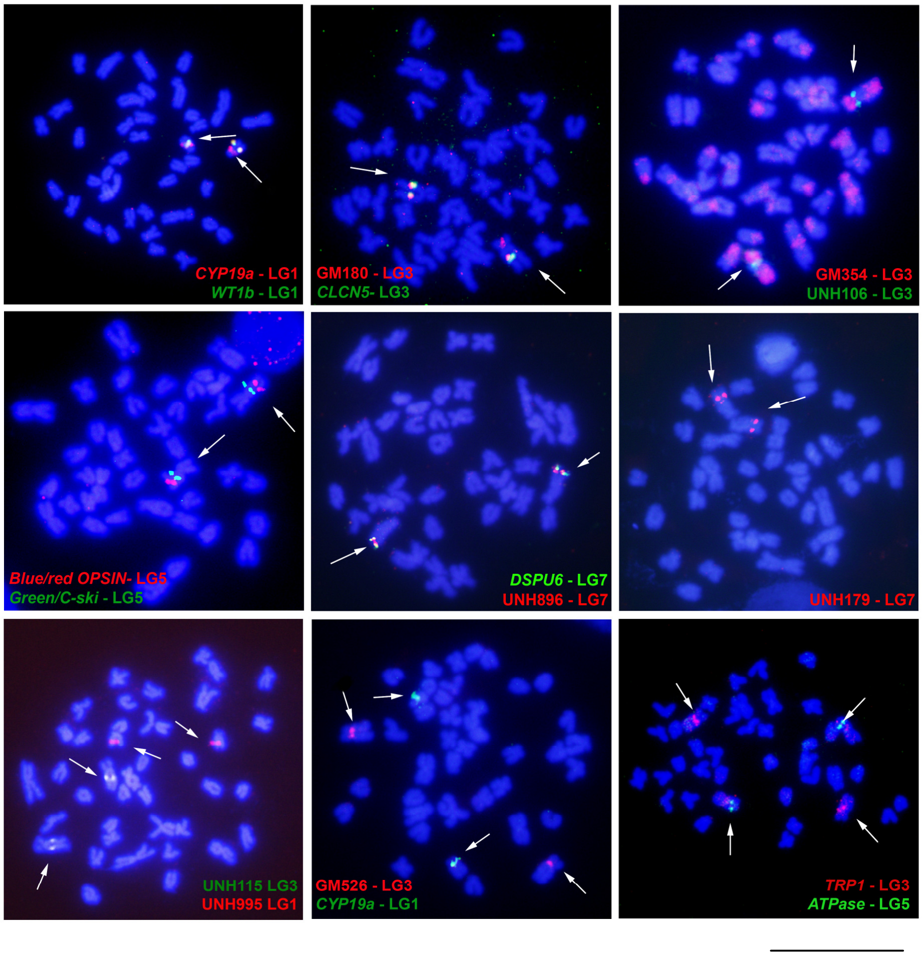

Labeotropheus trewavasae

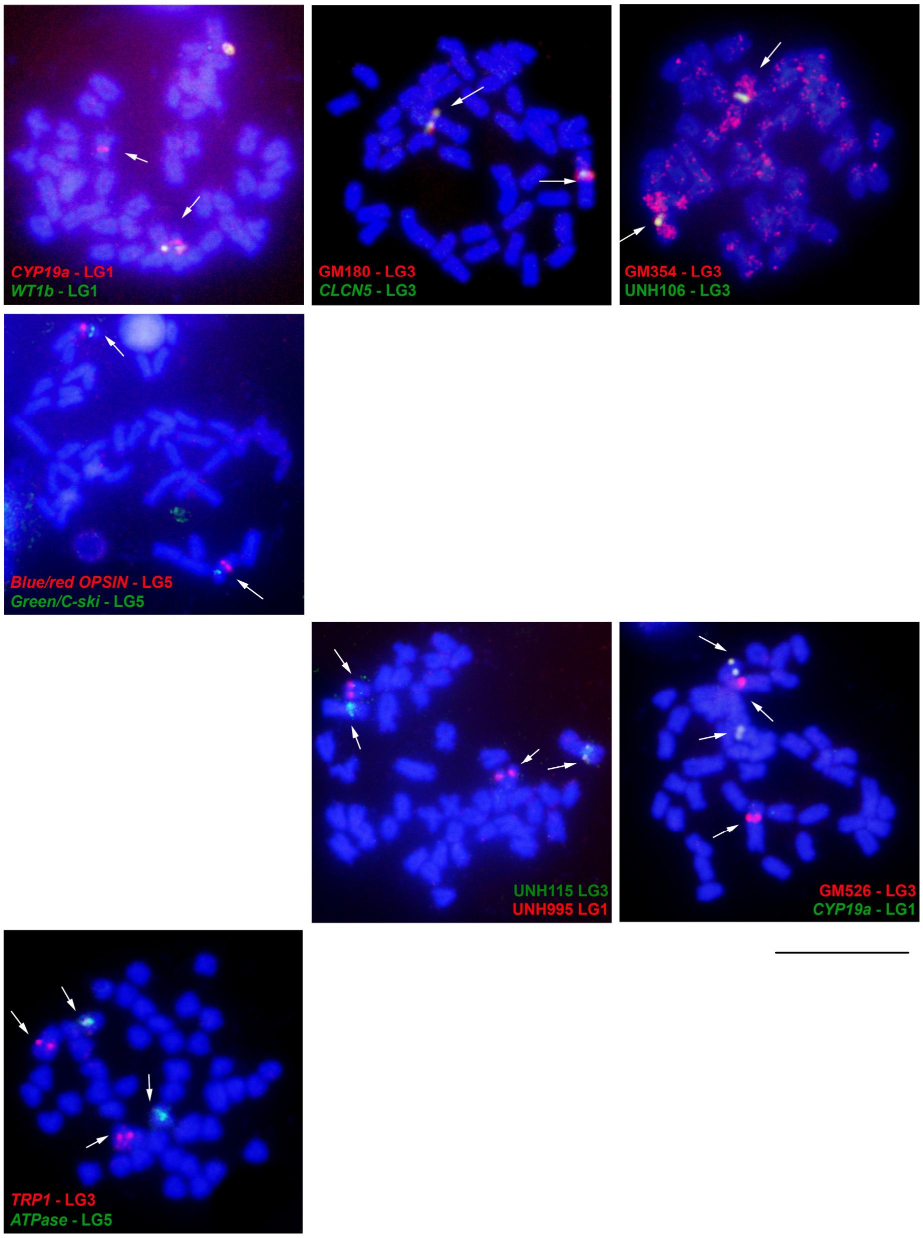

Hemichromis bimaculatus

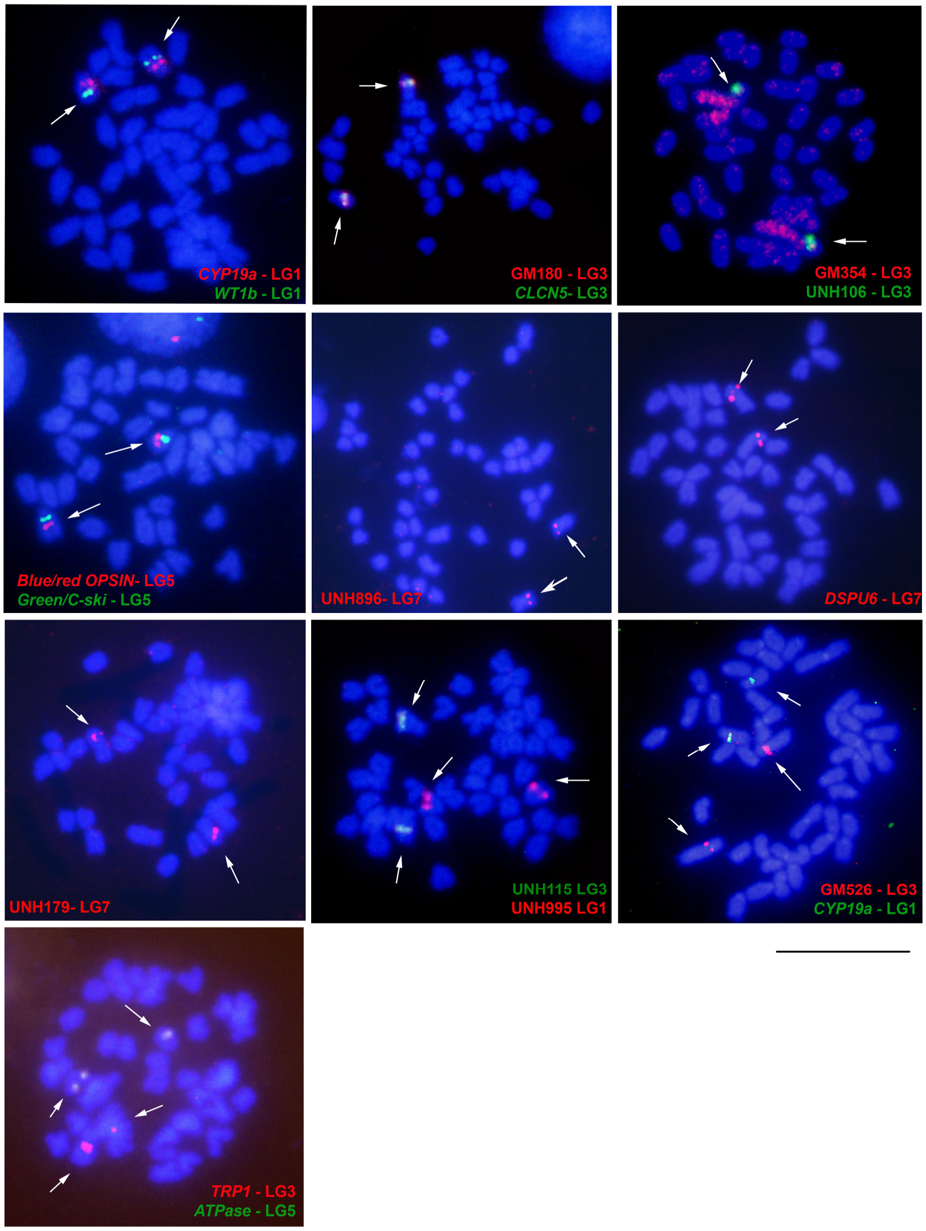

Supplement: Additional file 1 — Metaphase spreads of cichlid species probed with BAC clones fromOreochromis niloticus. BACs containing markers of LG1, 3, 5, and 7 were hybridized through FISH procedure and are indicated in different colors in each metaphase. The arrows indicate the chromosome position of probes. [file 1471-2164-13-463-S1.pdf]
