# Supplementary material for: Integrating cytogenetics and genomics in comparative evolutionary studies of cichlid fish
Source: BMC Genomics. 2012 Sep 9;13:463. doi: 10.1186/1471-2164-13-463 (PMC3463429; doi:10.1186/1471-2164-13-463)
Supplement: Additional file 2 — Genomicus/Ensembl accession numbers. Datasheet with accession numbers and markers of all analyzed sequences in Genomicus/Ensembl database for the LG1, 3, 5, and 7. Analyzes were conducted in July 2011. [file 1471-2164-13-463-S2.pdf]

Additional file 2:

Datasheet with accession numbers and markers of all sequences analyzed in Genomicus/Ensembl database for the LG1, 3, 5 and 7.

Analyzes were conducted in September 2011.

Major group/Species	Markers of LG1		Markers of LG3
	CYP19a	Wt1a/b	CLCN5
**Mammals**			
Homo sapiens - Human	ENSG00000137869	ENSG00000184937	ENSG00000171365
Pan troglodytes - Chimpanzee	ENSPTRG00000007074	ENSPTRG00000003476	ENSPTRG00000021899
Pongo abelii - Orangutan	ENSPPYG00000006475		ENSPPYG00000020349
Callithrix jacchus - Marmoset		ENSCJAG00000005900	ENSCJAG00000002945
Macaca mulatta – Rhesus monkey	ENSMMUG00000002553	ENSMMUG00000009881	ENSMMUG00000023056
Canis familiaris - Dog	ENSCAFG00000015338	ENSCAFG0000000742	ENSCAFG00000015971
Bos taurus - Bovine	ENSBTAG00000014890		ENSBTAG00000001507
Equus cabalus - Horse	ENSECAG00000020474	ENSECAG00000022308	ENSECAG00000010745
Sus scrofa - Pig		ENSSSCG00000013316	
Oryctolagus cuniculus - Rabbit	ENSOCUG00000012303	ENSOCUG00000005882	ENSOCUG00000017336
Rattus norvegicus – Rat	ENSRNOG00000000196	ENSRNOG00000013074	ENSRNOG00000002862
Mus musculus – Mouse		ENSMUSG00000016458	ENSMUSG00000004317
Monodelphis domestica - Opossum		ENSMODG00000009646	ENSMODG00000011303
**Birds**			
Gallus gallus – Chicken	ENSGALG00000013294	ENSGALG00000012115	ENSGALG00000007234
Meleagris gallopavo - Turkey		ENSMGAG00000001489	ENSMGAG00000004934
Taeniopygia guttata – Zebra finch	ENSTGUG00000006993	ENSTGUG00000004906	ENSTGUG00000005324
**Reptiles**			
Anolis carolinensis - Lizard		ENSACAG00000002480	
**Fish**			
Danio rerio – Zebrafish	ENSDARG00000041348	ENSDARG00000007990 / ENSDARG00000031420	ENSDARG00000019693 / ENSDARG00000022466
Oryzias latipes - Medaka	ENSORLG00000002949		ENSORLG00000008602
Tetraodon nigroviridis - Pufferfish	ENSTNIG00000009520		
Gasterosteus aculeatus – Stickleback			

Major group/Species	Markers of LG5				
	**AKR**	**Mme**	**ATPase**	**Opsin**	**C-ski**
**Mammals**					
Homo sapiens - Human	ENSG00000117448	ENSG00000196549	NM_001160234.1	ENSG00000102076	ENSG00000157933
Pan troglodytes - Chimpanzee	ENSPTRG00000000676	ENSPTRG00000015550	XM_513679.3	ENSPTRG00000022427	ENSPTRG00000000047
Pongo abelii - Orangutan	ENSPPYG00000001410	ENSPPYG00000014224	NM_001133855.1	ENSPPYG00000020867	
Callithrix jacchus - Marmoset		ENSCJAG00000000959	XM_002751283.1		
Macaca mulatta – Rhesus monkey	ENSMMUG00000020085	ENSMMUG00000006662	XM_001112641.2	ENSMMUG00000006166	ENSMMUG00000010280
Canis familiaris - Dog		ENSCAFG00000008711	XM_850193.1	ENSCAFG00000019441	ENSCAFG00000019387
Bos taurus - Bovine	ENSECAG00000000223	ENSBTAG00000002075		ENSBTAG00000020299	ENSBTAG00000038716
Equus cabalus - Horse	ENSECAG00000006082	ENSECAG00000009138		ENSECAG00000018684	ENSECAG00000012543
Sus scrofa - Pig	ENSSSCG00000003491	ENSSSCG00000011723			
Oryctolagus cuniculus - Rabbit	ENSOCUG00000014622	ENSOCUG00000017924			
Rattus norvegicus – Rat	ENSRNOG00000017780	ENSRNOG00000009514	NM_012506.1	ENSRNOG00000037261	
Mus musculus – Mouse	ENSMUSG00000028743	ENSMUSG00000027820	BC037206.1	ENSMUSG00000031394	ENSMUSG00000029050
Monodelphis domestica - Opossum	ENSMODG00000015881	ENSMODG00000015805		ENSMODG00000025355	
**Birds**					
Gallus gallus – Chicken	ENSGALG00000004005	ENSGALG00000010331		ENSGALG00000002848/19	ENSGALG00000001229
Meleagris gallopavo - Turkey	ENSMGAG00000005392	ENSMGAG00000010779		ENSMGAG00000007057	ENSMGAG00000002204
Taeniopygia guttata – Zebra finch		ENSTGUG00000011304		ENSTGUG00000007365	ENSTGUG00000002944
**Reptiles**					
Anolis carolinensis - Lizard		ENSACAG00000003903		ENSACAG00000012605	
**Fish**					
Danio rerio - Zebrafish	ENSDARG00000016649	ENSDARG00000026398	NM_131687.1	ENSDARG00000044862	ENSDARG00000042151/ ENSDARG00000008034
Oryzias latipes - Medaka	ENSORLG00000004388	ENSORLG00000005790		ENSORLG00000014058	ENSORLG00000003949
Tetraodon nigroviridis - Pufferfish	ENSTNIG00000014801	ENSTNIG00000007690		ENSTNIG00000007322	ENSTNIG00000014776
Gasterosteus aculeatus – Stickleback		ENSGACG00000010305			

Major group/Species	Markers of LG7			
	**RERG**	**KCNE1L**	**DUSP6**	**IGFBP2**
**Mammals**				
Homo sapiens – Human	ENSG00000134533	ENSG00000176076	ENSG00000139318	ENSG00000115457
Pan troglodytes – Chimpanzee	ENSPTRG00000004731		ENSPTRG00000005282	
Pongo abelii – Orangutan	ENSPPYG00000004331	ENSPPYG00000020626	ENSPPYG00000004817	
Callithrix jacchus – Marmoset	ENSCJAG00000021602	ENSCJAG00000023434	ENSCJAG00000019653	
Macaca mulatta – Rhesus monkey	ENSMMUG00000010460	ENSMMUG00000004187	ENSMMUG00000006227	ENSMMUG00000018940
Canis familiaris – Dog	ENSCAFG00000012854	ENSCAFG00000018048	ENSCAFG00000006100	ENSCAFG00000014490
Bos taurus – Bovine	ENSBTAG00000002174	ENSBTAG00000012319	ENSBTAG00000004587	ENSBTAG00000005596
Equus cabalus – Horse	ENSECAG00000014358		ENSECAG00000010089	ENSECAG00000012058
Sus scrofa – Pig	ENSSSCG00000000602	ENSSSCG00000012582		
Oryctolagus cuniculus – Rabbit	ENSOCUG00000000650	ENSOCUG00000005007	ENSOCUG00000002902	ENSOCUG00000008561
Rattus norvegicus – Rat	ENSRNOG00000027592		ENSRNOG00000023896	ENSRNOG00000016957
Mus musculus – Mouse	ENSMUSG00000030222	ENSMUSG00000090122	ENSMUSG00000019960	ENSMUSG00000039323
Monodelphis domestica – Opossum	ENSMODG00000017938		ENSMODG00000020516	ENSMODG00000015540
**Birds**				
Gallus gallus – Chicken	ENSGALG00000013078	ENSGALG00000023613	ENSGALG00000011207	ENSGALG00000011469
Meleagris gallopavo – Turkey	ENSMGAG00000013542		ENSMGAG00000011218	ENSMGAG00000011630
Taeniopygia guttata – Zebra finch				ENSTGUG00000005113
**Reptiles**				
Anolis carolinensis - Lizard	ENSACAG00000011754		ENSACAG00000011871	
**Fish**				
Danio rerio - Zebrafish	ENSDARG00000041104		ENSDARG00000070914	ENSDARG00000052470/ ENSDARG00000031422
Oryzias latipes - Medaka	ENSORLG00000009225			ENSORLG00000014695
Tetraodon nigroviridis - Pufferfish	ENSTNIG00000006917		ENSTNIG00000016051	ENSTNIG00000008865
Gasterosteus aculeatus – Stickelback				
